# Switched-Biasing Techniques for CMOS Voltage-Controlled Oscillator

**DOI:** 10.3390/s21010316

**Published:** 2021-01-05

**Authors:** Cheol-Woo Kang, Hyunwon Moon, Jong-Ryul Yang

**Affiliations:** 1 Department of Electronic Engineering, Yeungnam University, Gyeongsan, Gyeongbuk 38541, Korea; cwkang@yu.ac.kr; 2 Department of Electronic Engineering, Daegu University, Gyeongsan, Gyeongbuk 38453, Korea

**Keywords:** CMOS, voltage-controlled oscillator, switched-biasing, flicker noise, phase noise, current source, figure-of-merit

## Abstract

A voltage-controlled oscillator (VCO) is a key component to generate high-speed clock of mixed-mode circuits and local oscillation signals of the frequency conversion in wired and wireless application systems. In particular, the recent evolution of new high-speed wireless systems in the millimeter-wave frequency band calls for the implementation of the VCO with high oscillation frequency and low close-in phase noise. The effect of the flicker noise on the phase noise of the VCO should be minimized because the flicker noise dramatically increases as the deep-submicron complementary metal-oxide-semiconductor (CMOS) process is scaled down, and the flicker corner frequency also increases, up to several MHz, in the up-to-date CMOS process. The flicker noise induced by the current source is a major factor affecting the phase noise of the VCO. Switched-biasing techniques have been proposed to minimize the effect of the flicker noise at the output of the VCO with biasing AC-coupled signals at the current source of the VCO. Reviewing the advantages and disadvantages reported in the previous studies, it is analyzed which topology to implement the switched-biasing technique is advantageous for improving the performance of the CMOS VCOs.

## 1. Introduction

A voltage-controlled oscillator (VCO) is a key component in a frequency synthesizer that generates local oscillator (LO) signals for frequency conversion in a radio-frequency (RF) transceiver [1,2,3]. A VCO-based readout circuit, which is that the output voltage of the sensing core is applied to the node of the VCO tuning voltage, has merit to achieve a low sensitivity level and a high signal-to-noise ratio compared to the amplifier-based readout circuit [4,5]. In addition, high integration and low power consumption of the VCO-based readout circuit are advantageous for implementing a large-scale sensor array [6,7]. Radar sensors that monitor the change of electromagnetic-wave between the transmitted and received signals generated from the VCO remotely measure the distance, velocity, and vital-signs in real time [8,9,10]. Various sensors using VCOs require low phase noise characteristics in VCOs [11,12,13]. The frequency synthesizer such as the phase-locked loop (PLL) is conventionally used to reduce the phase noise, but the noise characteristics still remain at the output signal of the frequency synthesizer because the loop bandwidth of the PLL in the synthesizer is generally determined to be between 100 and 500 kHz [14,15].

A complementary metal-oxide-semiconductor (CMOS) process is a standard fabrication technology to implement electrical circuits; it can integrate control, logic, analog, and RF circuits into a single-chip system [16]. Moreover, the recent CMOS device designed in the several nanometer-scale shows competitive performances in transconductance (*g_m_*) and minimum noise figure (*NF_min_*), compared to the compound semiconductor device [17]. However, the transistor device implemented in the up-to-date CMOS process exhibits an increase in the flicker noise, which intrinsically depends on the physical structure of the channel and the flicker corner frequency, where the magnitudes of the flicker and white noises present equal increases up to several MHz or more [18,19]. The flicker noise is called “1/f noise” because the noise is increased as the frequency in the channel decreases [20]. The nanometer-scaled CMOS technology has advantages such as high integration, low power consumption, and high operating frequency, but it has the disadvantage of noise deterioration in the low-frequency band owing to the increase in the flicker noise [14].

The reduction in the flicker noise is a major issue in VCO design using the CMOS process because the phase noise of the VCO is mainly determined by the noise in the low-frequency region [21]. Many studies have been conducted to improve the reduction in the CMOS VCO phase noise performance caused by the flicker noise. Biasing techniques for core transistors have been widely used to prevent the degradation of the VCO phase noise by the flicker noise of the oscillator core transistors [22,23]. A VCO using core transistors biased at class C operation is a representative technique that reduces the contribution of the flicker noise effect from the core transistor at the output [24,25,26]. A resonant filter at the second harmonic also minimizes the noise effect from the core transistors, but the large chip size and the tuning range limit cause other issues in the design of the VCO using this technique [27]. The increase in the chip area can be reduced by implementing the filter using the common-mode resonance of the LC tank in a cross-coupled LC oscillator, although the issue of the tuning range limit cannot be solved [15,28,29]. The performance degradation by the noise of the core transistors is reduced by these techniques, but the contribution of the flicker noise caused by the current source, which is used to constantly supply the DC bias current in the core transistors, remains in the output characteristics of the VCO. The flicker noise by the current source dramatically increases the close-in phase noise of the VCO owing to the nonlinear characteristics of the VCO [30]. A simple method to reduce the effect of the flicker noise from the transistors constituting the current source is to design the VCO using only voltage biasing, that is, without using any current sources [14,31]. However, the core current of the VCO can easily deviate from the designed value depending on the power supply variations when current biasing is not used. It can also be observed that the oscillator becomes more sensitive to ground noise [21]. A switched-biasing technique has been proposed to reduce the flicker noise effect of the current source based on the periodic behavior of the differential VCO [32,33]. The up-conversion behavior of the flicker noise of the current source can be fundamentally eliminated using the switched-biasing technique, which is based on the periodic operation of the differential VCO [34].

In this paper, we review operations, features, and implementation examples of the VCO using the switched-biasing technique as a method to reduce the flicker noise effect generated by the current source. The effect of improvement in the phase noise by this technique is examined based on a comparison of the VCO performance obtained by the topologies implementing switched-biasing. In particular, it is analyzed whether the switched-biasing technique is useful for generating the millimeter-wave signals, which are increasingly used in various applications. Section 2 introduces the first proposal of a switched-biasing technique for the reduction of the flicker noise effect of CMOS transistors and describes the advantages of the technique in the VCO design. In Section 3, the switched-biasing technique is classified into three topologies based on how the biasing voltage of the current source is configured, and the features and implementation examples of VCOs using each topology are presented. Section 4 discusses the advantages and disadvantages of each topology by comparing the CMOS VCOs with switched-biasing techniques. Based on the discussion results, the applicability of the switched-biasing technique for the millimeter-wave signal generation, which is mandatory to the high-resolution radar sensors operating at 20 GHz or more, is examined.

## 2. Switched-Biasing Technique

As the deep-submicron CMOS process is scaled down, the low-frequency noise (especially the flicker noise) of the MOSFET becomes more important in the design of CMOS RF transceivers. It has long been known that the flicker noise is generated in a variety of homogeneous semiconductor bulks and is observed in various devices, such as a vacuum tube, diode, and MOSFET [35,36]. Various research works have been conducted to identify the cause of the flicker noise and to clearly understand its characteristics clearly [32,33,35,36,37,38,39,40,41,42,43]. To predict the flicker noise phenomenon generated in MOSFETs, Hooge published a carrier mobility fluctuation (CMF) model, in which the flicker noise is caused by the mobility fluctuation of free carriers in the device [37]. McWhorter suggested a carrier number fluctuation (CNF) model, where the low frequency noise of the MOSFET is generated by the fluctuation in the number of charge carriers in the device [38]. The two presented models were useful for understanding the physical mechanism of the flicker noise, but their limitation is that they can only be applied to the long-channel devices. The flicker noise in short-channel devices is mainly considered to be due to the random telegraph signal (RTS) noise generated by the Si−SiO_2_ interface because as the size of the devices is scaled down, the device operation is predominantly represented by the movement of each charge carrier [39,41,44].

**Figure 1:**
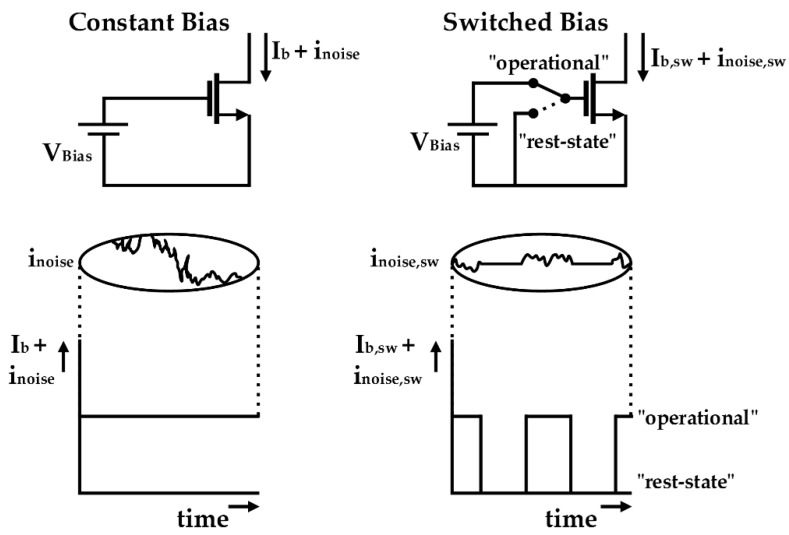
Conceptual diagram of switched-biasing technique (reproduced with permission from the author, reducing MOSFET 1/f noise and power consumption by switched biasing; published by IEEE, 2000) [33].

Research on reducing the intrinsic flicker noise of MOSFETs began in the early 1990s. Bloom and Nemirovsky first suggested that the flicker noise of the MOSFET could be reduced by cycling between inversion and accumulation of the device [40]. They explained that the device noise in the on-state can be reduced when the off-state exists before the on-state. Dierickx and Simoen revealed that the flicker noise reduction by inversion-to-accumulation cycling is related to the emptying of traps at the interface that generates RTS noise [41]. Based on the principle of inversion-to-accumulation cycling, Gierkink et al. proposed a switched-biasing technique [32]. Figure 1 shows the operating principle of the switched-biasing technique [33]. The “operational state” in Figure 1 means that the MOSFET operates at the inversion state, to facilitate the flow of current between the drain and the source. The drain–source current does not flow at the “rest-state” of the MOSFET because the bias voltage at the gate is lower than the threshold voltage of the device. A reduction in the flicker noise can be expected by the periodic operation between the two states of the device and is verified with a simple mathematical analysis. Assuming a duty cycle of 50%, the drain–source current of the MOSFET by the switching operation can be expressed as the multiplication of the flicker noise and a square-wave signal *m*(*t*) with the duty cycle,

(1) $$m(t)=\frac{1}{2}+\frac{2}{\pi }\sin(\omega_{sw}t)+\frac{2}{3\pi }\sin(3\omega_{sw}t)+\frac{2}{5\pi }\sin(5\omega_{sw}t)+\cdots ,$$

where *ω_sw_* is the angular frequency of the switching operation. Because the power spectral density (PSD) of the noise in the low-frequency band is determined by the convolution of the DC component of *m*(*t*) and the flicker noise, the switched-biasing technique can decrease the PSD by 6 dB compared to the constant-biasing technique. In addition, several studies have confirmed that the flicker noise is further reduced when the transistor is sufficiently turned off (i.e., deep accumulation). This reduction is known to be caused by the elimination of the long-term-memory effect associated with the flicker noise [32,33,44]. The analysis of the operating characteristics and principle shows that the PSD due to low-frequency noise depends on the bias state at the time and the bias history in a periodic operation [44]. This phenomenon was verified in both NMOS and PMOS because the carrier type does not affect the operating principle [43].

**Figure 2:**
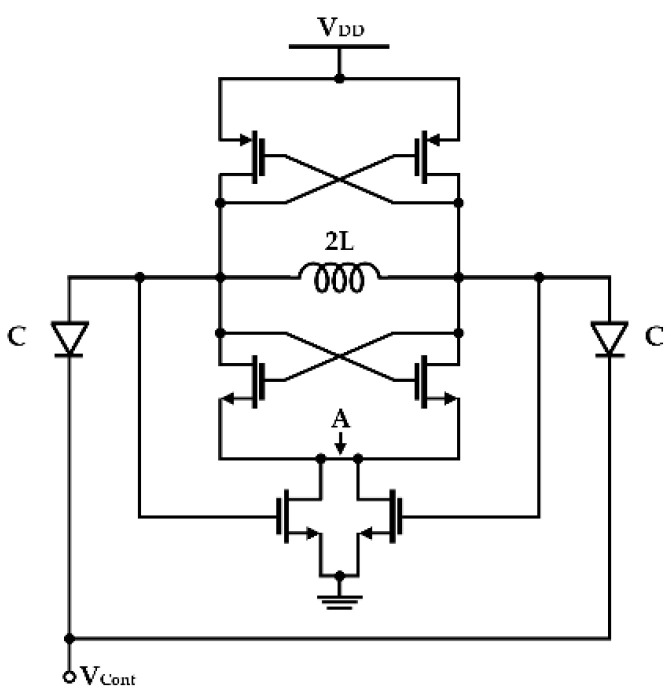
Schematic of a CMOS LC-VCO with the switched-biasing technique to the current source (reproduced with permission from the author, RF CMOS low-phase-noise LC oscillator through memory reduction tail transistor; published by IEEE, 2004) [34].

Flicker noise reduction by using the switched-biasing technique has been applied to various circuit designs, such as amplifiers and frequency mixers requiring low-noise characteristics [32,33,34,45,46]. In particular, the switched-biasing technique can be useful for the oscillator to improve the performance because the output signal in the oscillator is generated by the periodic switching operation of the transistor. After Gierkink et al. showed that the effect of flicker noise in the ring oscillator can be reduced by applying the switched-biasing technique to the DC bias control, Kluperink et al. demonstrated improvement in closed-in phase noise by switching the current source in the sawtooth oscillator [33]. Boon et al. showed that the switched-biasing technique can improve the phase noise of the LC oscillator by reducing the up-conversion of the flicker noise of the current source [34]. As shown in Figure 2, the switched-biasing of the current sources is implemented by the output oscillation signals, and the DC level of the current sources is self-biased by the structural characteristics of a CMOS LC-VCO. The VCO shown in Figure 2 shows phase noise improvement of 6 dB and 3 dB at 10 kHz offset compared to the VCO with the fixed-biasing current source and the VCO without the current source, respectively [34].

**Figure 3:**
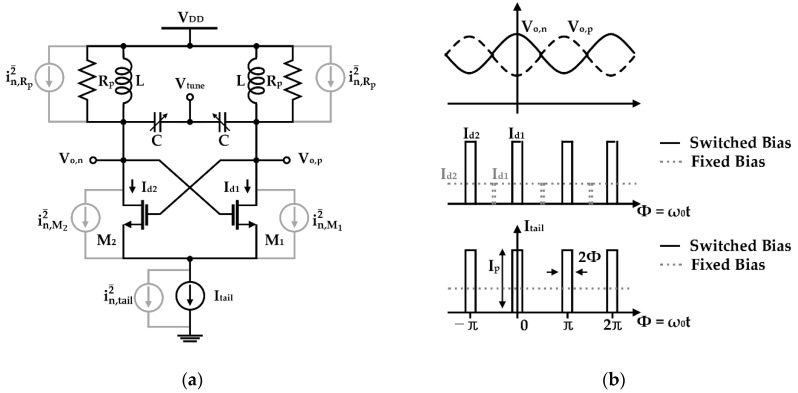
Analysis of VCO characteristics depending on the pulse modulation of the current source using the impulse sensitivity function theory: (**a**) schematic of the differential LC-VCO; (**b**) conceptual waveforms of the output voltages and drain currents of the VCO and the bias current by the pulse-modulated current source (reproduced with permission from the author, Tail current-shaping to improve phase noise in LC voltage-controlled oscillators; published by IEEE, 2006) [47].

The characteristics that the current source modulated by the switched-biasing technique is effective in improving the phase noise of a VCO was proved using a theoretical analysis based on a mathematical model [47]. The proposed theoretical analysis is based on the impulse sensitivity function (ISF) theory, which can explain the phase noise contribution depending on the output voltage swing of the VCO [48]. The proposed analysis in Figure 3 shows that the phase noise of a VCO can be greatly improved by additionally injecting the bias current to the VCO core transistors at the time when the voltage swing of the VCO is maximized or minimized. This phenomenon is based on the fact that the time when the output voltage of the VCO becomes the maximum or minimum has the minimum sensitivity to the phase shift [47]. Figure 3b shows that the phase noise of the VCO can be minimized by the modulation signals of 2*f*_0_ in the current source compared to the fixed-biasing current source. It is caused that the bias currents of the cross-coupled transistors in the VCO using the switched-biasing current source are limited at a time of high phase-shift sensitivity and supplied at a time of low phase-shift sensitivity. The currents *I_d_*_1_ and *I_d_*_2_ supplied from the switched biasing current source are not supplied at the highly sensitive time in the phase noise where the output voltages *V_o_*_,*n*_ and *V_o_*_,*p*_ are crossed. Based on physical and theoretical interpretations, it can be verified that the switched-biasing technique improves the phase noise of the VCO by modulating the current source.

## 3. Circuit Implementation

There are various design approaches to implement the switched-biasing technique for current source modulation in the VCO. Three design specifications in the current source should be considered in the implementation of the VCO with this technique:

- Modulation frequency and amplitude
- Modulation waveform
- DC bias voltage

The oscillation frequency of the VCO or the specific frequency generated from an external signal source can be used as the modulation frequency, which is related to the amount of flicker noise reduction [44,49,50]. The modulation amplitude should be sufficiently large to ensure periodic inversion-to-accumulation operations at the current source [51,52]. The modulation waveform affects whether the current source operates as hard-switching or soft-switching and the efficiency of the noise reduction [47]. A DC bias voltage should be determined as the specific value (e.g., the threshold voltage of the transistor constituting the current source) to obtain the effective switching operation, considering the modulation amplitude [52,53,54]. Based on these specifications, the proposed VCO design methods using the switched-biasing technique are divided into three topologies. Depending on whether the individual source for bias modulation is used or not, they are largely divided into external-biasing and self-biasing topologies. The self-biasing topology is further subdivided according to the usage of fixed or adaptive DC bias voltages. The detailed implementation methods of the switched-biasing technique applied to various VCO architectures are shown within the classification of these three topologies.

### 3.1. External-Biasing Topology

To verify the effectiveness of the flicker noise reduction phenomenon, in the initial study, the gate bias of the MOS device was externally applied [32,40,41]. Similarly, the switched-biasing technique can be implemented by externally controlling the gate bias of the current source of the VCO. In addition, the efficiency of the flicker noise reduction by the switched-biasing technique can be significantly improved in the VCO because the external signal generator can be optimally designed with the modulation frequency, amplitude, waveform including the duty cycle, and DC bias voltage.

**Figure 4:**
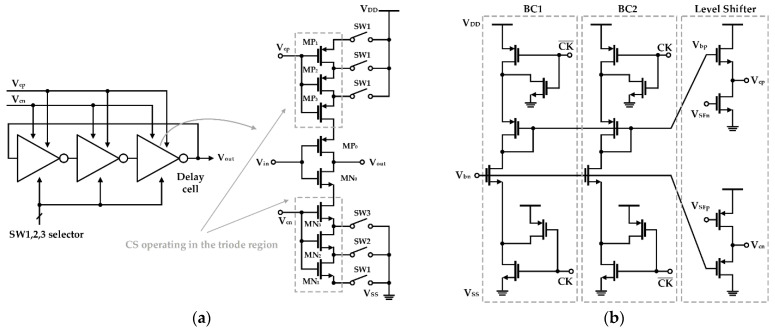
Ring VCO based on a switched-bias circuit (SBC): (**a**) block diagram of the ring VCO and schematic of the delay cell in the VCO; (**b**) schematic of SBC for current source modulation (reproduced with permission from the author, Low-voltage, low-phase-noise ring voltage-controlled oscillator using 1/f-noise reduction techniques; published by The Japan Society of Applied Physics, 2007) [50].

Yoshida et al. proposed a structure that digitally controls the current source of a ring-type VCO using a switched-bias circuit (SBC), which is depicted in Figure 4 [50]. As shown in Figure 4b, the SBC is composed of two-level shifters and two identical bias branches (BC1 and BC2) and is configured to operate alternately according to the clock (CK) signal applied from the outside. In the VCO core shown in Figure 4a, current sources divided into three bits are placed on each side of the delay cell, and their gate bias is switched to a modulated signal (V_cp_ and V_cn_) formed through the SBC. In addition, all current sources are biased to perform a triode operation because the probability of trap−detrap is less than that of the saturation operation and less flicker noise is generated [55]. The clock frequency of the SBC was set to 10 MHz. The purpose is to suppress spurious occurrences at the switching frequency despite the simulation result that noise reduction below 100 kHz is independent of the clock frequency. Effectively utilizing the SBC, the ring VCO improves the noise performance by 3 dB at 100 kHz [50].

### 3.2. Self-Biasing Topology with the Fixed DC Voltage

A fully on-chip-type circuit cannot be configured by externally applying the modulation signal, and the design of the switched-bias technique is complicated because of the spurious dependence of the modulation frequency [50,56]. Although the external-biasing topology can supply the optimal modulation signal to the current source, the modulation signal correlated with the oscillation signal cannot be guaranteed to have an additional current injection at the time with the minimum sensitivity to the phase shift, as shown in Figure 3b. The phase noise of the oscillator can be further reduced by driving the current source modulated with the frequency which is the same as the oscillation frequency [34,47].

**Figure 5:**
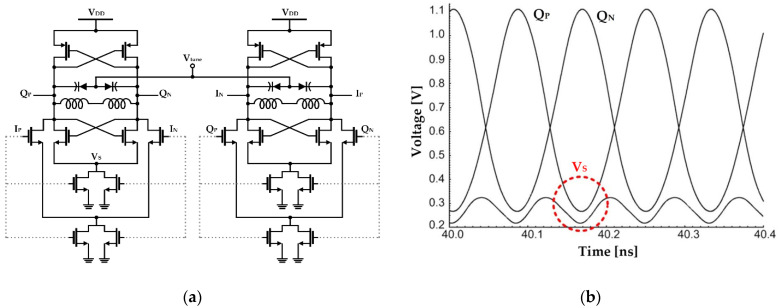
CMOS quadrature-VCO (QVCO) with separate current sources based on the switched-biasing technique: (**a**) schematic of the QVCO; (**b**) simulation waveforms at the outputs of the quadrature channel and the common node (reproduced with permission from the author, Low-phase-noise CMOS quadrature VCO; published by IEEE, 2006) [57].

Jeong and Yoo proposed a method of applying switched-biasing to the current sources of each *g_m_* stage and the coupled input stage in a CMOS quadrature-VCO (QVCO), as presented in Figure 5 [57]. When applied to a conventional current source coupled QVCO, the switched-biasing technique can be applied to the current source shared by the coupled input stage and the *g_m_* stage. However, when the *g_m_* stage and the coupled input stage share a current source, the oscillation waveform and the common source node waveform are misaligned because of the resistance in the transistor triode region and parasitic capacitances in the common source node (shown as Vs in Figure 5a). In this design, the source nodes of the cross-coupled pair and the coupled-input pair are separated for optimal alignment, and the QVCO waveform is shown in Figure 5b. As a result, the oscillation amplitude increased by 0.3 V (peak-to-peak) compared to the structure that shared the current source. Compared to the constant bias current and shared-current source method, phase noise improvements of 17 dB and 10 dB were shown in the simulation, respectively. In the measurement results, an improvement in the performance of 10 dB by the switched-biasing technique was verified, compared to that of the shared-current source method [57].

**Figure 6:**
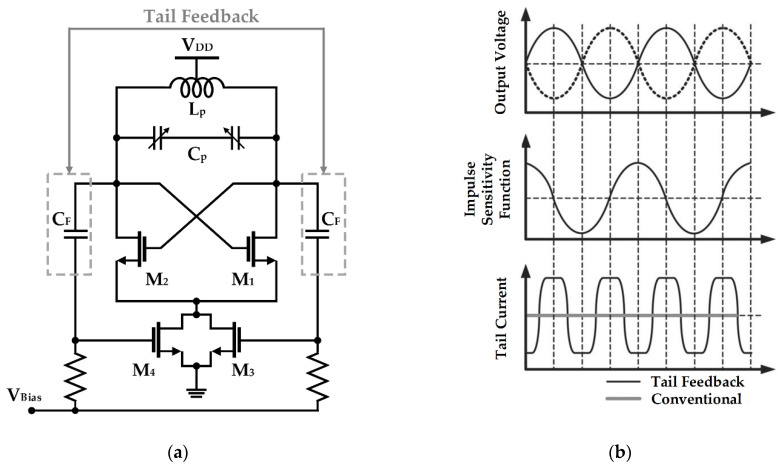
Millimeter-wave NMOS VCO using the switched-biasing technique: (**a**) schematic of the VCO; (**b**) waveform analysis of the VCO based on the ISF theory (reproduced with permission from the author, a low phase noise quadrature injection locked frequency synthesizer for mm-wave applications; published by IEEE, 2011) [51].

Musa applied switched-biasing to the current source of a VCO operating near millimeter-wave, as shown in Figure 6 [51]. Unlike the CMOS structure, as it is an NMOS-only structure, an additional bias path (as shown in V_Bias_ of Figure 6a) for setting an appropriate DC bias level and a capacitor (as shown in *C_F_* of Figure 6a) for coupling with the oscillation node are added. As depicted in Figure 6b, based on the ISF theory, the phase noise was improved through optimal current injection (i.e., the zero crossing point of the ISF) [47]. In addition, as the size of the feedback capacitance *C_F_* determines the modulation signal amplitude, the capacitance was determined as an optimal value considering the trade-off between phase noise improvement and power consumption [51].

**Figure 7:**
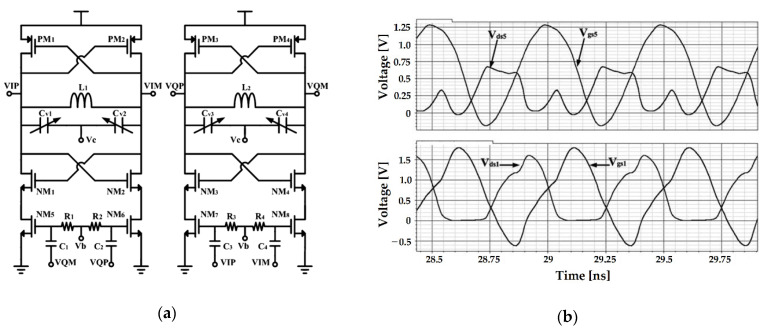
CMOS QVCO with split current sources: (**a**) schematic of the QVCO; (**b**) simulated waveforms at the drain–source and gate-source voltages of NM5 (top) and NM1 (bottom) (reproduced with permission from the author, Low phase noise self-switched biasing CMOS LC Quadrature VCO; published by IEEE, 2009) [52].

Huang and Kim proposed a self-biasing QVCO using the current source splitting (CSS) method, as illustrated in Figure 7a [52]. Unlike the conventional method of sharing a current source, it is designed to separate and deliver current to each cross-coupled transistor. This method has the advantage of being able to ignore noise caused by parasitic capacitance appearing at the common source node of a cross-coupled pair through separation of the corresponding node. In addition, the current source (NM_5−6_ in Figure 7a) and the cross-coupled pair (NM_1−2_ in Figure 7a) act as two cascode cross-coupled pairs, creating an effective negative resistance. As mentioned in Section 2 and as shown in Figure 7b, the long-term memory effect was eliminated by maximizing the modulation amplitude (i.e., VCO in the voltage-limited region), thus increasing the flicker noise reduction effect of the current source. To prove this, the result of the circuit simulation with which the flicker noise factor of the MOSFET was removed as compared to the measurement result of the fabricated QVCO, and similar phase noise improvement was confirmed [52].

**Figure 8:**
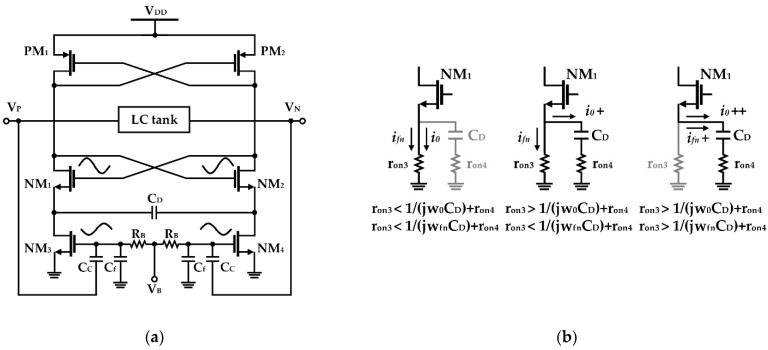
CMOS VCO including degeneration and filter capacitors: (**a**) schematic of the VCO; (**b**) current flows depending on the capacitance of CD—the usage of the optimal capacitance can divide the current into two paths, as shown in the middle case (reproduced with permission from the author, Reduction of 1/f^3^ phase noise in LC oscillator with improved self-switched biasing; published by Springer, 2015) [58].

Chen et al. proposed a method to suppress the flicker noise generated from cross-coupled pairs by adding a source degeneration capacitor, as depicted in Figure 8a [58]. The degeneration capacitor (*C_D_* in Figure 8a) is set to have a low impedance at the fundamental frequency and high impedance at a low frequency (i.e., flicker noise), as presented in Figure 8b [59]. In addition, using a filtering capacitor (as shown in *C_f_* of Figure 8a), a low pass filter was constructed to remove noise from the bias path. In the simulation results, the closed-in phase noise of the VCO was improved by 2 dB using the current source modulation, but the structure using the degeneration capacitor improved 4, 11, and 7.5 dB at 10 kHz, 100 kHz, and 1 MHz, respectively [58].

**Figure 9:**
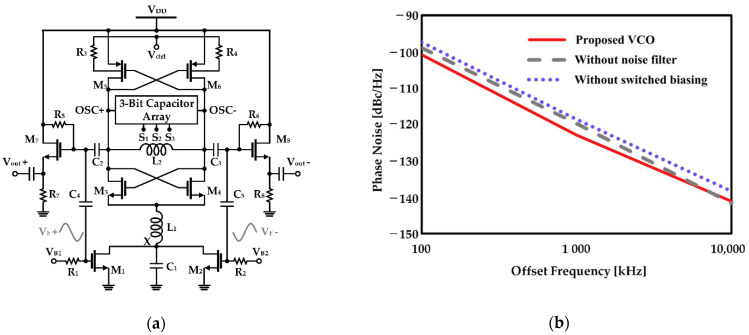
CMOS VCO with improved phase noise characteristics by adding a passive network: (**a**) schematic of the VCO; (**b**) phase noise of the VCO with the current source modulation and the noise filter (displayed as “proposed VCO”), the VCO using only the current source modulation (displayed as “without noise filter”), and the VCO using the constant bias voltage without the filter (displayed as “without switched biasing”) (reproduced with permission from the author, A 0.7-mW LC voltage-controlled oscillator leveraging switched biasing technique for low phase noise; published by IEEE, 2019) [53].

Hsieh and Lin proposed adding a passive network between the current source and the VCO core to suppress the up-conversion of the second harmonic noise, as shown in Figure 9a [53]. As the second harmonic current of the common source node of the cross-coupled pair is up-converted and acts as noise, *C*_1_, shown in Figure 9a, is connected in parallel with the current source to filter the second harmonic thermal noise [27]. Moreover, as the quality-factor (Q-factor) of the LC tank decreases during the period when the cross-coupled pair transistor operates in the triode, *L*_1_ is added to increase the impedance of the common source node. As shown in Figure 9b, it was verified that the closed-in phase noise (100 kHz−1 MHz) characteristic improved to 3 dB when the passive network was added based on the same switched-biasing technique [53].

**Figure 10:**
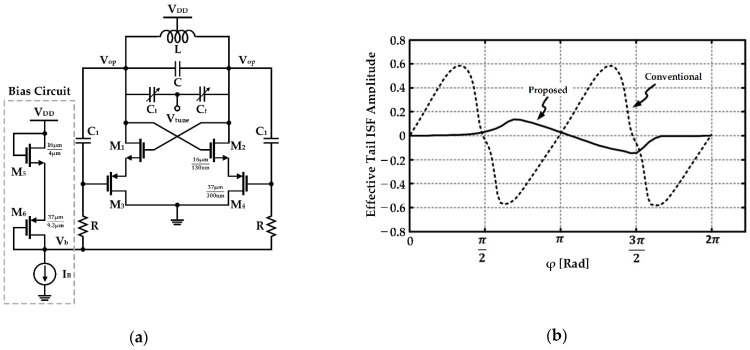
CMOS VCO based on the ISF manipulation technique: (**a**) schematic of the VCO including current sources using PMOS transistors; (**b**) waveforms of the effective tail ISF amplitude for the conventional current source using NMOS and the proposed current source using PMOS (reproduced with permission from the author, a 2.4 GHz VCO with FOM of 190dBc/Hz at 10 kHz-to-2 MHz offset frequencies in 0.13 µm CMOS using an ISF manipulation technique; published by IEEE, 2015) [60].

Based on the ISF theory, Mostajeran et al. proposed the ISF manipulation technique to reduce the flicker noise contribution by reducing the effective ISF of the tail current source, as shown in Figure 10 [60]. Considering that the ISF in the current source, it is necessary to implement two turn-offs during one oscillation period, and a separated current source structure was adopted in a similar manner to previous studies. By deep triode operation of the PMOS transistor as a current source, a low impedance path to the ground is formed so that less noise generated from the tail flows into the tank. Owing to switched biasing and the operation of the triode region of the PMOS, it can be observed that the effective ISF of the current source is reduced compared to the conventional NMOS current source, as depicted in Figure 10b. The measurement indicated an improvement of 17 dB in phase noise at 10 kHz and 8.2 dB at 1 MHz compared to phase noise with the structure using the conventional NMOS current source, and a very low flicker corner frequency of 10 kHz was confirmed [60].

**Figure 11:**
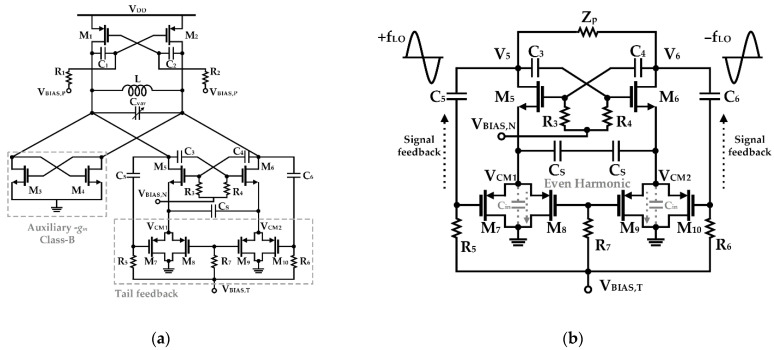
Class-C CMOS VCO using the switched-biasing technique: (**a**) schematic of the VCO; (**b**) bias conduction at the sub-threshold operation of the split current sources implemented by PMOS transistors (reproduced with permission from the author, A 2.2 to 2.9 GHz complementary class-C VCO with PMOS tail-current-source feedback achieving—120 dBc/Hz phase noise at 1 MHz offset; published by IEEE, 2019) [54].

Shasidharan et al. proposed a structure in which the switched-biasing technique is applied to a Class-C CMOS VCO, as shown in Figure 11 [54]. In this design, a source degeneration capacitor to suppress flicker noise of a cross-coupled pair and an auxiliary −*g_m_* stage to compensate for insufficient negative *g_m_* were constructed. A current source using PMOS transistors was used to make a low impedance path, and an effective switching operation was achieved by the biasing at the sub-threshold voltage. Moreover, by adjusting the size of the current source appropriately, the parasitic capacitance *C_in_* was designed to be an even-mode harmonic filter of the common node (V_CM1−2_ in Figure 11b). As it was designed to have a narrow conduction angle of 0.31π through simulation, the phase noise characteristic shows an improvement of 14 dB in the performance at 1 MHz offset compared to the case where the switched-biasing technique was not applied [54].

**Figure 12:**
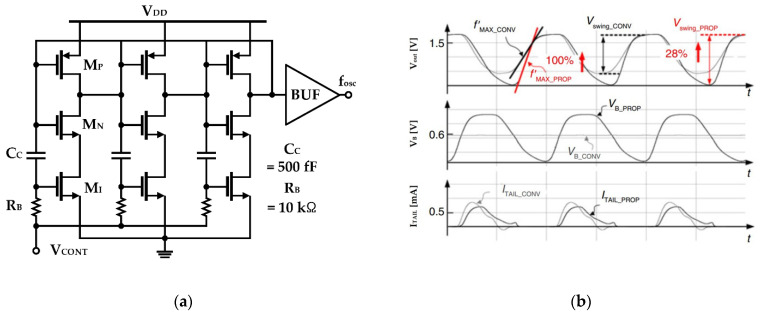
Ring VCO using the switched-biasing technique: (**a**) schematic; (**b**) simulated waveforms of the VCO output (top), bias voltage of the current source (middle), and tail current of the inverter cell (bottom) (reproduced with permission from the author, Low phase noise ring VCO employing input-coupled dynamic current source; published by IET, 2020) [61].

Lee and Im applied the switched-biasing technique in a simple inverter delay cell based ring oscillator, which is depicted in Figure 12 [61]. As shown in Figure 12b, by self-biasing the current source of the CMOS inverter, the slope of the output waveform increases, and, as a result, the flicker noise is reduced and the oscillation swing is improved [62]. Compared to the topology without self-biasing, an improvement of 7 dB at 100 kHz and 11.5 dB at 1 MHz was verified through simulation [61].

As the self-bias topology is coupled from the oscillation node, there is the advantage that an external modulation signal is not required. In addition, because it is implemented in the design of the VCO alone, the noise design can be controlled differently from the case of external bias. However, in some studies, the DC bias level is set near the threshold voltage of the current source for a proper switching effect **[53,54,63]**. Unsurprisingly, switching of the current source occurs after oscillation has begun, and, hence, an excessively low DC bias may not provide adequate starting conditions.

### 3.3. Self-Biased Topology with the Adaptive DC Voltage

To solve the start-up issue of the general self-bias topology, a method of adaptively adjusting the DC bias level of the current source according to the oscillation amplitude can be used. As mentioned in Section 2, because the current efficiency can be improved by reducing the conduction angle, a narrow conduction angle can be implemented by lowering the DC bias level as it approaches the steady state.

**Figure 13:**
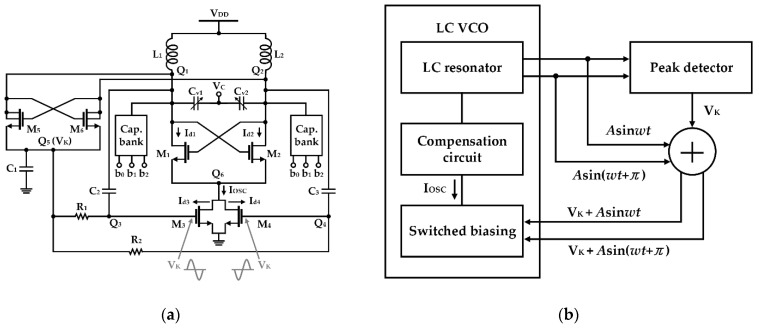
VCO using self-biasing with the adaptive DC voltage: (**a**) schematic; (**b**) operation of the self-biasing with the adaptive DC voltage from peak tracking at the oscillator outputs (reproduced with permission from the author, Low voltage CMOS LC VCO with switched self-biasing; published by Wiley, 2009) [63].

Min et al. suggested that the DC bias level of the current source can be adaptively adjusted according to the VCO oscillation amplitude by adding an auxiliary peak detector to the existing self-biasing topology, as shown in Figure 13 [63]. In this design, referring to Figure 13b, a separate cross-coupled pair (depicted in M_5_−M_6_ of Figure 13a) detects the negative peak of the oscillation waveform and induces charging to the capacitor (as shown in *C*_1_ in Figure 13a). The charged voltage (*V_k_*) changes the DC bias level of the current source, solving the start-up issue of the oscillator, and simultaneously reducing the oscillator amplitude variability, mainly due to the Q-factor change of the capacitor bank within the tuning range and the process, voltage, and temperature (PVT) variation of the circuit. The phase noise using the switched-biasing technique was improved to approximately 6 dB at 100 kHz in the simulation, compared to that using the constant-biasing technique [63].

**Figure 14:**
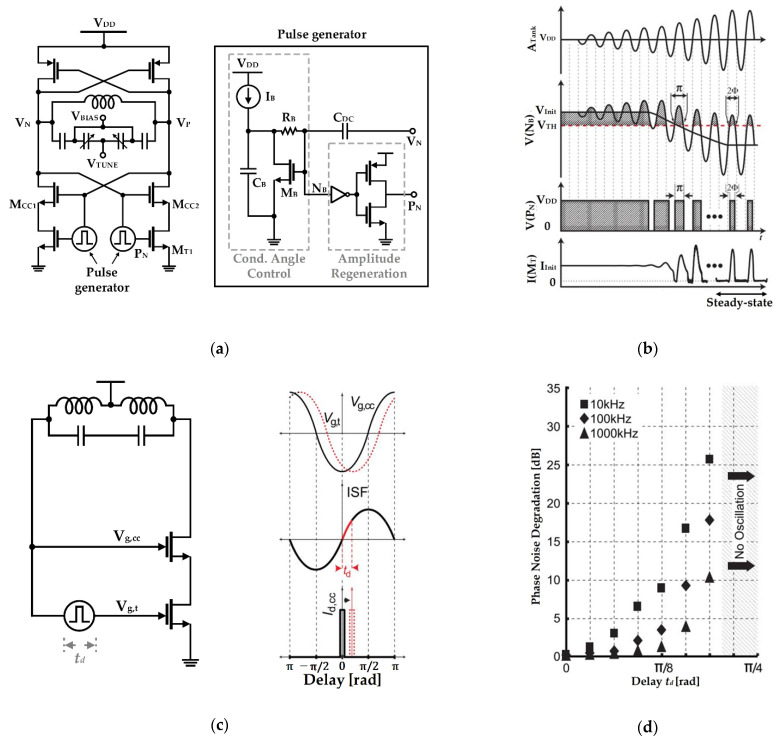
VCO employing the pulse generator that supplies pulse waveforms and the adaptive DC bias voltage: (**a**) schematic; (**b**) conceptual diagram of the VCO including the pulse generator operation; (**c**) intrinsic delay of the pulse generator; (**d**) simulated phase noise degradation with increasing the delay of the pulse generator (reproduced with permission from the author, A pulse-tail-feedback LC-VCO with 700 Hz flicker noise corner and −195dBc FoM; published by IEICE, 2019) [64].

Narayanan and Okada presented the VCO architecture using a synchronized pulse generator to inject pulse-shaped waveforms into each current source, as shown in Figure 14 [64]. Unlike the self-biasing with the fixed DC voltage in which a modulation signal is injected directly through a capacitor, a rail-to-rail waveform is implemented using a two-stage inverter to narrow the conduction angle. As an additional method to reduce the conduction angle, an envelope tracking scheme called conduction angle control, shown in Figure 14a, was used. It lowers the DC bias level applied to the current source as the VCO oscillates, as indicated in Figure 14b, so that the conduction angle decreases as the VCO reaches a steady state. As the DC bias is approximated to the supply voltage when the current source supplies current, the triode operation is possible; thus, the noise generation of the current source is reduced compared to the case of the saturation operating point. Because of this design method, this VCO lowered the flicker noise corner to 700 Hz, and thus obtained the result of having a flattened figure-of-merit (FoM) in the range of 1 kHz−10 MHz. However, the intrinsic delay of the pulse generator clarifies the limitations of this method, as shown in Figure 14c. For proper current bias based on the ISF theory, a positive peak voltage must be delivered to the current source at the point where the ISF is zero crossing, but an indispensable mismatch occurs because of the corresponding delay. The result is shown in detail in Figure 14d. By adjusting the delay of the pulse waveform modeled with Verilog-A, when the delay exceeds 8/π, the phase noise degradation occurs rapidly, and even when it reaches 4/π, it can be observed that oscillation does not occur [64].

## 4. Discussion

The switched-biasing technique in which the current source of the VCO is modulated with AC signals can reduce the closed-in phase noise of the VCO to minimize the generation of the flicker noise in the source. However, the effect on the overall performance of the VCO differs depending on the implementation method of generating the AC modulating signal. There are two general methods to generate the modulating signal: one uses the external signal generator and the other uses the oscillation signal coupled with the output of the VCO.

**Figure 15:**
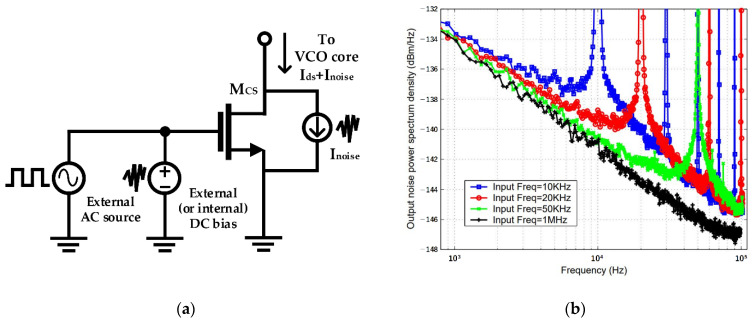
Characteristic of external-biasing topology: (**a**) conceptual schematic of external biasing (also available in PMOS configuration); (**b**) large noise peaks appearing as harmonics of the modulation frequency correlated with the external signal (reproduced with permission from the author, Experimental study on MOSFET’s flicker noise under switching conditions and modelling in RF applications; published by IEEE, 2001) [56].

The external-biasing topology, which is a method using an external signal generator, showed that the closed-in phase noise of the VCO can be improved by the AC modulating signal of the current source [32,50]. However, it has drawbacks in the implementation of the integrated circuit because the chip area, power consumption, and design complexity can be increased by providing an external signal generator. In addition, as shown in Figure 15a, it is difficult to show the improvement in the performance caused by the reduction of the closed-in phase noise because the induced noise from an external generator can directly result in performance degradation of the VCO. The performance degradation caused by the induced noise from the external generator may be greater than the performance improvement caused by the reduction in the closed-in phase noise. The low correlation between the AC modulation signal and the oscillation signal may also increase the noise contribution of the external generator at the output of the VCO, as depicted in Figure 15b. It has been reported that the noise reduction of the VCO with an external generator is independent in the band below the modulation frequency, but the noise signals that are dependent on the modulation frequency are presented at the output of the VCO [42,49,50,56]. Based on the results of previous studies, it can be understood that the modulation frequency of the current source in the switched-biasing technique should be set to a frequency that does not affect the phase noise of the VCO. For example, the modulation frequency can be set to a frequency over the flicker corner frequency.

**Figure 16:**
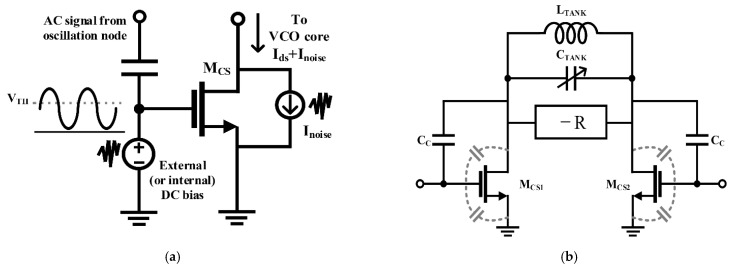
Characteristics of the self-biasing topology: (**a**) description of the self-biasing; (**b**) tuning range limit by the effect of the parasitic capacitances present at the current sources.

The self-biasing topology, which is a method using the coupled signal from the VCO output, has been proposed to solve the problem caused by the use of an external generator [34]. As the tail current source of the VCO in the self-biasing topology is modulated by the oscillation signal, the correlation between the modulation signal and the output can be achieved using easy implementation without additional circuitry. The current efficiency driving the VCO core can be increased by decreasing the conduction angle owing to the correlated AC modulation signal of the current source, based on the ISF theory [47,48]. In addition, the effect of the flicker noise of the current source is dramatically reduced in the self-biasing topology by the modulation frequency in the GHz band, which is higher than the flicker corner frequency. The DC bias voltage of the current source becomes an important design condition when the switched-biasing technique applies to the NMOS VCO using a low power supply voltage of 1.2 V or less. The efficiency of the switching operation is generally determined by the DC bias voltage, which is set near the threshold voltage because the current modulation should be exhibited by a small-sized switching signal. The DC bias of the current source in the initial research stage using the switched-biasing technique was set to the same biasing state as before the AC modulation of the source. However, the bias was changed to the threshold voltage of the current source transistor for clearly switching the on-off states of the current flow due to AC modulation [53,54,63]. The DC bias near the threshold voltage may not sufficiently supply the driving current for VCO operation, because the biasing current is generated at the sub-threshold region of the current source transistor, as shown in Figure 16a. In addition, it is difficult to apply to commercial circuits as operation reliability problems of the current source may occur owing to the PVT variation. Because the thermal noise produced from the voltage source for setting the DC bias can degrade the phase noise of the VCO, a method for reducing the contribution of the thermal noise should be applied to the circuit design for supplying the DC bias [58]. The capacitance of the resonator in the VCO is increased by the output coupling lines for self-biasing, as depicted in Figure 16b, and the resonance frequency of the LC tank can be affected by this increase. The coupling capacitor *C_C_* in Figure 16b, which determines the amplitude of the modulation signal, should be generally designed to be higher than the parasitic capacitance of the current source [51,58]. The frequency shift due to the additional capacitances becomes an important factor in designing the high-frequency VCO because the total reactance in the LC tank decreases as the oscillation frequency increases. Above all, the major problem in DC biasing near the threshold voltage of the current source is that the current source does not operate in the saturation, and the common node of the VCO does not achieve a high impedance. Low impedance at the common node may cause the deduction of the phase noise as more noise from the switching operation of the current source affects the VCO core [58]. The effect on impedance reduction at the common node by DC biasing near the threshold voltage may be minimized as proposed by the previous studies, which include the method of splitting the current source into several transistors, the method of using a source degeneration capacitor, and the method of implementing an additional filter for noise reduction [52,54,58,60,64].

When the DC biasing of the current source is set to a voltage higher than the threshold voltage, the flicker noise may not be reduced by degrading the effect of the switching operation at the current source. An adaptive DC biasing technique that sets the biasing voltage differently depending on the amplitude of the oscillation signal has been proposed to reduce the operation problem generated by the fixed DC biasing at the current source. The adaptive biasing technique has the advantage of increasing the stability of the VCO operation and the robustness of the start-up operation. It was shown that the self-biasing topology implementing adaptive DC biasing using a negative peak detector can compensate for PVT variation and the variation of the Q-factor of the varactor that occurs in tuning the oscillation frequency [63]. A self-biasing topology with a pulse generator with three different operating states has been proposed to achieve a low conduction angle in steady state and a fast start-up time [64]. As the main drawback, the auxiliary circuit to implement the adaptive DC biasing requires additional loading to reduce the Q-factor of the LC tank and tuning range and increases the power consumption. The advantages and disadvantages of each topology implementing the switched-biasing technique are summarized in Table 1.

**Figure 17:**
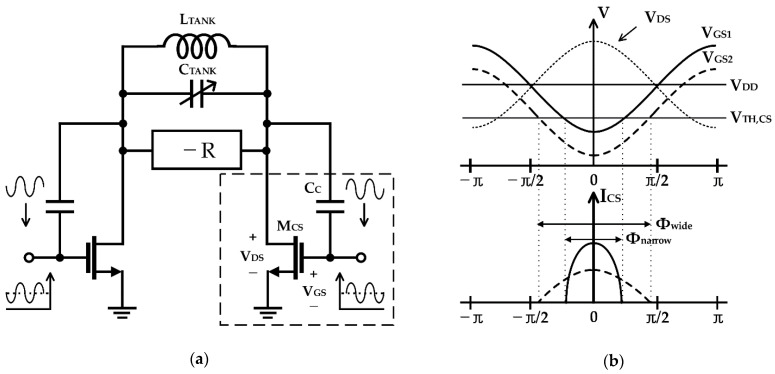
Conduction angle in the VCO operation: (**a**) description of the conduction angle in the VCO; (**b**) comparison of the conduction angle between the constant-biasing and the switched-biasing techniques.

The performances of the CMOS VCO using the switched-biasing technique are summarized in Table 2. The performances of VCOs oscillating at different frequencies are quantitatively compared using the conventional figure-of-merit (*FoM*) and the figure-of-merit with tuning range (*FoM_T_*) as follows [65]:

(2) $$FoM[dBc]=L(\Delta f)-20\log\left(\frac{f_{0}}{\Delta f}\right)+10\log\left(\frac{P_{DC}}{1mW}\right),$$

(3) $$FoMT[dBc]=L(\Delta f)-20\log\left(\frac{f_{0}}{\Delta f}\right)+10\log\left(\frac{P_{DC}}{1mW}\right)-20\log\left(\frac{TR}{10}\right),$$

where *L*(∆*f*) is the phase noise of the VCO in dB at the frequency offset ∆*f* from the center oscillation frequency, *P_DC_* is the power consumption, and *TR* is the frequency tuning range in Hz. The phase noise, *FoM*, and *FoM_T_* are normalized at a frequency offset of 1 MHz. In the ring-VCO, the self-biasing topology showed more improvement in the phase noise and *FoM* than the external-biasing topology. The LC-VCOs using the self-biasing topology showed a relatively high level of *FoM* below −179 dBc and *FoM_T_* below −174 dBc. The LC-VCO with a *FoM* of −190 dBc showed a *FoM_T_* of −179 dBc, which is a relatively low performance owing to the narrow tuning range [60]. The high *FoM* of −190 dBc is based on the phase noise reduction by the switched-biasing technique along with the reduction in the intrinsic noise and current consumption by using a PMOS current source operating in the triode mode [54,60]. The high *FoM* and *FoM_T_* of the VCO with the adaptive DC voltage, high modulation amplitude, and pulse waveform in the self-biasing topology show that the minimum conduction angle can be useful for improving the VCO performance [64]. Figure 17 shows that the performance of the VCO can be improved by reducing the noise injection when the modulation waveform is implemented as a pulse with a duty cycle of less than π in the switched-biasing technique [64].

## 5. Conclusions

It was shown with physical and theoretical analyses that the switched-biasing technique can improve the phase noise characteristics by modulating the current source in the VCO using the deep sub-micron CMOS process. The switched-biasing technique can be divided into external-biasing and self-biasing topologies, depending on the method of implementing the current modulation. Even though the external-biasing topology can apply an optimum waveform as the modulation signal, the self-biasing topology that can control the current source with a waveform correlated with the output signal shows higher improvement in performance. The self-biasing topology can be subdivided into the usage of a fixed DC voltage and an adaptive DC voltage. The self-biasing topology with an adaptive DC voltage can be expected to apply the optimized waveform to the modulation signal; however, there is no significant improvement in the VCO performance compared to the self-biasing topology with a fixed DC voltage because the implementation of additional circuits, which are required for the adaptive DC voltage, increases the noise injection to the VCO. In addition, the self-biasing topology with the adaptive DC voltage is not also suitable for the millimeter-wave VCO design because the additional circuits can increase the parasitic components that affect the oscillation frequency shift, tuning range limit, and design accuracy. Based on the improvement of the phase noise, ease of implementation, and overall *FoM* and *FoM_T_*, it could be concluded that the self-biasing topology with a fixed DC voltage is the most useful in the switched-biasing technique.

## Figures and Tables

**Table 1:** Characteristics of different bias schemes.

Topologies	Bias Techniques	Advantages	Disadvantages
External-biasing	- The current source’s gate is biased by external clock generator.	- Effectively reducing flicker noise generation.	- Requires circuit or equipment to generate ac signal. - Uncorrelated ac source noise directly degrades phase noise.
Self-biasing with the fixed DC voltage	- The current source’s gate is coupled with the oscillation node of VCO. - DC bias is fixed to a specific value.	- No additional AC source required. - Circuit noise controlled internally	- Thermal noise is introduced through bias path. - Start-up issue
Self-biasing with the adaptive DC voltage	- The current source’s gate is coupled with the oscillation node of VCO. - DC bias is controlled by the feedback path.	- VCO amplitude robust to PVT variation - Prevent oscillation start-up issue	- Complex circuitry - The adaptive DC biasing circuit is loaded directly to the VCO core, degrading performance.

**Table 2:** Performance summary of CMOS VCO using a switched-biasing technique.

Ref. (year)	Process (µm)	Bias Scheme	VCO Type	V_DD_ (V)	Freq. (GHz)	Tuning Range (%)	Phase Noise @1 MHz (dBc/Hz)	Power (mW)	*FoM* @1 MHz (dBc/Hz)	*FoM_T_* @1 MHz (dBc/Hz)
[50]	0.18	External biasing	CMOS	1	1	82	−88 ^1^	0.71	−149 ^1^	−168 ^1^
(2007)			Ring VCO							
[61]	0.18	Self-biasing w. fixed DC	CMOS	1.8	1	47.6	−106	1.2	−165	−179
(2020)			Ring VCO							
[51]	0.065	Self-biasing w. fixed DC	NMOS	1.2	20	17	−107	19.2	−179	−184
(2011)			LC-VCO							
[58]	0.18	Self-biasing w. fixed DC	CMOS	1.2	2.55	9.2	−122.8	3.2	−186	−185
(2015)			LC-VCO							
[60]	0.13	Self-biasing w. fixed DC	NMOS	1.4	2.4	1.7	−128.4	4.2	−190	−174
(2015)			LC-VCO							
[54]	0.18	Self-biasing w. fixed DC	CMOS	1.2	2.45	28.6	−120	1.73	−185	−195
(2019)			LC-VCO							
[53]	0.18	Self-biasing w. fixed DC	CMOS	0.8	1.4	18	−123	0.7	−187	−193
(2019)			LC-VCO							
[57]	0.13	Self-biasing w. fixed DC	CMOS	1.2	5	20	−117	5.28	−184	−190
(2006)			LC-QVCO							
[52]	0.18	Self-biasing w. fixed DC	CMOS	1.8	2	17	−134.5	36	−185	−190
(2009)			LC-QVCO							
[63]	0.13	Self-biasing	NMOS	0.6	4.85	10.2	−117	3.9	−185	−185
(2009)		w. adaptive DC	LC-VCO							
[64]	0.18	Self-biasing	CMOS	1.2	4.55	4.3	−123.4	1.35	−195	−188
(2019)		w. adaptive DC	LC-VCO							

^1^ Normalized at 1 MHz for comparison.

## Data Availability

No new data were created in this study. Data sharing is not applicable to this article.
